# Sildenafil, a Type-5 Phosphodiesterase Inhibitor, Fails to Reverse Myeloid-Derived Suppressor Cell-Mediated T Cell Suppression in Cells Isolated From Tuberculosis Patients

**DOI:** 10.3389/fimmu.2022.883886

**Published:** 2022-07-22

**Authors:** Vinzeigh N. Leukes, Stephanus T. Malherbe, Andriette Hiemstra, Leigh A. Kotze, Kelly Roos, Alana Keyser, Dalene De Swardt, Andrea Gutschmidt, Gerhard Walzl, Nelita du Plessis

**Affiliations:** ^1^ DSI-NRF Centre of Excellence for Biomedical Tuberculosis Research, South African Medical Research Council for Tuberculosis Research, Division of Molecular Biology and Human Genetics, Faculty of Medical and Health Sciences, Stellenbosch University, Cape Town, South Africa; ^2^ Division of Medical Virology, Department of Pathology, Institute of Infectious Disease and Molecular Medicine, University of Cape Town, Cape Town, South Africa; ^3^ Central Analytical Facility, Stellenbosch University, Cape Town, South Africa

**Keywords:** tuberculosis, *Mycobacterium tuberculosis*, host-directed therapies, myeloid-derived suppressor cells, Sildenafil

## Abstract

Successful TB treatment is hampered by increasing resistance to the two most effective first-line anti-TB drugs, namely isoniazid and rifampicin, thus innovative therapies focused on host processes, termed host-directed therapies (HDTs), are promising novel approaches for increasing treatment efficacy without inducing drug resistance. We assessed the ability of Sildenafil, a type-5 phosphodiesterase inhibitor, as a repurposed compound, to serve as HDT target, by counteracting the suppressive effects of myeloid-derived suppressor cells (MDSC) obtained from active TB cases on T-cell responsiveness. We confirm that MDSC suppress non-specific T-cell activation. We also show that Sildenafil treatment fails to reverse the MDSC-mediated suppression of T-cell functions measured here, namely activation and proliferation. The impact of Sildenafil treatment on improved immunity, using the concentration tested here, is likely to be minimal, but further identification and development of MDSC-targeting TB host-directed therapies are warranted.

## Introduction

Tuberculosis (TB), a communicable disease caused by *Mycobacterium tuberculosis* (*M.tb*), is a major cause of ill health and one of the leading causes of death worldwide (1). TB is curable and preventable, however the current 6-month regimen for drug-sensitive TB only achieves an 85% success rate after strict adherence, leaving 1.5 million people sick (1). Successful TB treatment is further hampered by increasing resistance to the two most effective first-line anti-TB drugs, namely isoniazid and rifampicin (1). Moreover, time-consuming, and costly drug development pipelines contribute to these TB treatment challenges. Therefore, more effective, cheaper, and smarter drug discovery approaches represent promising solutions to these TB treatment challenges.

Drug repurposing is a strategy for identifying new uses for approved or investigational drugs that are outside the scope of the original medical indication (2). Drug repurposing has been accepted globally as being more rapid and cost-effective than traditional drug discovery approaches (3, 4). The recent idea of repurposed drugs serving as HDT adjunct to the standard TB treatment regimen, is increasingly appreciated (5, 6). Host-directed therapies aim to better equip the host immune system by allowing a reduction in tissue damage caused by infection-induced inflammation, greater efficacy in pathogen elimination at the site of disease and improved treatment success rates (7).

MDSC are a heterogeneous population of myeloid cells, arising during chronic inflammation, with the capacity to suppress T-cell functions (8, 9). MDSC can be divided into at least two subsets with distinct morphology and suppressive mechanisms: firstly, monocytic MDSC (M-MDSC), morphologically similar to monocytes, macrophages, and dendritic cells, expressing high levels of NO; and secondly, polymorphonuclear MDSC (PMN-MDSC), morphologically similar to granulocytes, expressing high levels of ROS (8–10). MDSC frequencies are increased in humans at TB diagnosis (11), returning to levels consistent with otherwise healthy controls following successful treatment (11, 12). Moreover, MDSC with immuno-modulatory and suppressive effects were shown to be present in the blood and lung components of TB patients (13). MDSC have also been demonstrated to be present in the periphery of nonhuman primate granulomas, and restricted access to the TB granuloma core (14). Furthermore, MDSC have been shown to suppress T-cell proliferation in mycobacterial infections (11, 13, 15–17), as well as phagocytose both BCG and H37Rv (18), in a murine model. This work suggests that MDSC have a dual role in TB disease: firstly, by suppressing T-cell function and secondly harboring *M.tb*. Considering the immunosuppressive properties of MDSC in TB, ablation of these cells represents a feasible target for investigation of potential HDT.

During active TB, pro-inflammatory immune responses are often robust but fail to contain bacterial proliferation, leading to tissue damage and non-productive inflammation (19). Adjunctive TB HDT treatment aims avoid the development of irreversible lung damage from non-productive inflammatory responses to thereby improve the quality of life of TB survivors (20, 21). We recently reviewed a repurposed compound, currently being investigated in oncology studies, for its potential translation to control inflammation in the context of TB, namely Sildenafil (6). Sildenafil, a phosphodiesterase-5 inhibitor (PDE-5i), has been FDA/EMA approved to treat pulmonary hypertension, cardiac hypertrophy, and erectile dysfunction by increasing intracellular concentrations of cyclic guanosine monophosphate (cGMP) (22, 23). Subsequently PDE-5 inhibitors have also been shown restore immune effects and have consistent benefits in the treatment of male genitourinary dysfunctions [including benign prostatic hyperplasia (24, 25), lower urinary tract symptoms (26), and Peyronie’s disease (27)], as well as neurologic dysfunctions [neurogenesis and recovery from stroke (28–33)], tissue and organ protection [antineoplastic agent (34) and gastrointestinal damage (35, 36)], cutaneous ulcerations [antiphospholipid syndrome (37), scleroderma (38, 39), and systemic sclerosis (40, 41)], transplant and reconstructive surgery (42–46), female genital dysfunctions [fertility and preeclampsia (47–52)], and diabetes [neuropathy and vasculopathy (53–55)].

Sildenafil downregulates MDSC immunosuppressive activity in oncology (56, 57). PDE-5i was tested in mice and shown to be immune restorative by reversing tumor-induced immunosuppression and inducing antitumor immunity that delayed tumor progression. In particular, Sildenafil has been shown to improve cancer therapy by upregulating T-cell numbers in tumors and increasing T-cell activation and T-cell interleukin (IL)-2 production (58). Serafini *et al.* have shown that Sildenafil downregulated MDSC in a mouse model, enhancing T- and B-cell dependent immune responses as well as increasing CD8 T-cell recruitment to the site of inflammation thereby restoring antitumor immunity (58). More recently, studies have shown an increase of MDSC in melanoma lesions, with an associated downregulation in T-cell activity (56, 57). Pharmacological inhibition of PDE-5 attenuated MDSC immunosuppressive function and significantly increased survival of tumor-bearing mice (56, 57). A case report described a patient with multiple myeloma who, after being treated with a PDE-5 inhibitor, experienced a durable anti-tumor immune response and clinical improvement from reduced MDSC function (59). The mechanism by which PDE-5 inhibition downregulates MDSC activity is through inhibition of ARG1 and NOS2 expression which have been shown to be critical in immune suppression (58).

PDE-5i have also been tested in laboratory models of *M.tb* infection/TB disease, however, the effect of PDE-5i on host immune responses, specifically MDSC levels and function, in the context of human TB remains unknown. Reports show that Sildenafil addition to standard TB therapy accelerated *M.tb* sterilization in the mouse lung by 1 month as compared with standard treatment alone (60). Thus, adjunct PDE-i together with anti-TB chemotherapy may help shorten treatment duration and improve treatment outcome.

This investigation is the first human study evaluating the *in vitro* effect of Sildenafil (Viagra), a PDE-5i, on MDSC phenotype and function at diagnosis of active TB disease in the peripheral blood (PB) compartment. The overarching goal of this investigation is to assess pharmacological HDT-driven changes in innate immunosuppressive profiles in the periphery. The discovery that Sildenafil neutralises MDSC immunosuppressive functions during TB disease would have clinical relevance, as PDE-5 inhibitors targeting MDSC are currently being tested as repurposed adjunct treatment to standard cancer immunotherapies.

## Materials and Methods

### Cohort

The study cohort consisted of HIV uninfected participants who were recently diagnosed with active pulmonary TB disease and were treatment naïve (n=7), participant demographics shown in **Table 1**. The participants were recruited by the Stellenbosch University Biomedical Research Institute Clinical Team from the Ravensmead, Uitsig, Elsiesriver, Adriaanse and Fisantekraal suburbs of Cape Town, South Africa, following the National TB Program treatment practices. The diagnosis of these individuals was confirmed by a positive sputum MGIT culture or GeneXpert, in addition to radiological evidence of active disease. Written informed consent was obtained from all participants involved in the study, by dedicated research personnel, and study approval was given by the Stellenbosch University Ethics Review Committee (N16/05/070). Sodium heparinised peripheral blood (18 mL) was collected from all individuals. All experiments were performed in accordance with relevant guidelines and regulations.

**Table 1:** Participant Demographics.

PARTICIPANT DEMOGRAPHICS		
	MALE	FEMALE
Cohort: Count (%)	5 (71.4%)	2 (28.6%)
: Median Age (Range)	47 (27-60)	45.5 (45-46)

7 Adult, HIV negative patients, recently diagnosed with active pulmonary TB disease.

### PBMC Isolation

Ficoll density centrifugation (400 x g for 25 min) occurred following blood draw. PBMC were collected and washed with 1 x PBS at 400 x g for 10 min, followed by a second wash with MACS buffer (Miltenyi, Aubern, USA) 400 x g for 10 min. PBMC were counted using a haemocytometer and cell viability was checked by Trypan Blue Staining (80uL 1x PBS, 10uL trypan blue, 10uL cell suspension) (**Appendix 1A**.) Isolated PBMC remained fresh and were used immediately for cell isolations/enrichments.

### Cell Isolations/Enrichments

MACS isolation using LS columns (Miltenyi, Aubern, USA) followed and MDSC were enriched by depleting CD3+ cells, negatively selecting for HLA-DR and positively selecting for CD33 (AC104,3E3 clone). We also enriched for CD3+ T cells and control monocytes (CD3-HLA+). MACS isolation flow diagram shown in **Appendix 2**. Purity was checked by Flow cytometry, per cell population from each participant (**Figure 2**), and cell viability was checked by Trypan Blue staining, **Appendix 1A**.

### Co-Culture Assays

The isolated cells (MDSC and control monocytes) were cultured at 2x10 (5) cells per well in RPMI + 10% FBS + 1% L-glut with or without 10uM Sildenafil, in the presence/absence of T cells (1 Tcell: 1 MDSC), under 2 conditions including Unstimulated and anti-CD2CD3CD28 bead activated T cells, for 48 h at 37°C with 5% CO2. Cell culture layout shown in **Appendix 3**. Brefeldin A was added at 43 h, to increase intracellular cytokine concentrations for Flow cytometry analysis. Culture supernatant was harvested at 48 h and stored at -80°C for future Multiplex (BioPlex 200, Thermofisher, Waltham, USA) experiments and the cell fraction was stained for flow cytometry. Cell viability at varying concentrations of Sildenafil treatment shown in **Appendix 1B**. Sildenafil concentration calculation shown in **Appendix 4**.

### Flow Cytometry

For cell surface and intra-cellular analyses, we made use of two 8-colour panels including markers for (1) evaluation of MDSC phenotype (CD1c-PE-Cy7 (L161), CD3-FITC (UCHT1), CD11b-PerCP (M1/70), CD14-PacBlue (M5E2), CD15-BV-510 (W6D3), CD16-APC-Cy7 (3G8), CD33-PE (WM53) and HLA-DR-APC (G46-6)) and (2) T-cell function (CD3-FITC (UCHT1), CD4-PerCP-Cy5.5 (L200), CD8-APC-Cy7 (SK1), CD45RO-PacBlue (UCHL1), CD62L-APC (DREG-56), CD69-BV510 (FN50), FoxP3-PE (259D/C7) and Ki67-PE-Cy7 (B56)). Titrations, Compensation and Fluorescence-minus-one (FMO) was performed prior to analysis, on the FACS Canto II, at the BD Flow Cytometry Unit at Stellenbosch University, Tygerberg Campus. Data was analysed using the third party FlowJo Software v10.8.0 using the gating strategies attached in **Appendix 5**.

### Multiplex Assay

Quantification of cytokine levels from culture supernatants was performed using a human 37-plex assay (R&D Systems Human Luminex Discovery Assay LXSAHM-37 plex assay, Minneapolis, USA), on the Bioplex 200 (Bio-Rad, California, USA). The panel comprised of Interleukin (IL)-1α, IL-1β, IL-1rα, IL-2, IL-3, IL-4, IL-5, IL-6, IL-7, IL-8, IL-10, IL12p70, IL-15, IL17, Cluster of Differentiation (CD)-40L, Epidermal Growth Factor (EGF), Eotaxin, Basic Fibroblast growth factor (FGF), FMS-like Tyrosine Kinase 3 ligand (FLT3L), Fracktalkine, Granulocyte-Colony Stimulating Factor (G-CSF), Granulocyte-Macrophage Colony Stimulating Factor (GM-CSF), Growth Related Oncogene alpha (GROα), GROβ, Interferon gamma (IFNγ), Interferon Gamma-Induced Protein (IP)-10, Macrophage-derived Chemokine (MDC), Monocyte chemoattractant protein (MCP)-1, MCP-3, Macrophage Inflammatory Protein (MIP)-1α, MIP-1β, Platelet-derived growth factor (PDGFAA), PDGFBB, Transforming Growth Factor alpha (TGFα), Tumour Necrosis Factor alpha (TNFα), TNFβ and Vascular Endothelial Growth Factor (VEGF). Measurements were examined using Bio-Plex Manager v.4.1.1. software against internal quality controls and dilutions of cytokine standard curves were prepared according to manufacturer’s instructions.

### cGMP ELISA

Quantification of cGMP concentration from MDSC culture supernatants was performed using a cGMP ELISA kit (Elabscience, E-EL-0083, Houston, Texas, United States of America) according to the manufactures instructions.

### Statistical Analyses

The experimental design made use of paired testing of self-controlled samples before and after treatments and stimulations. We compared multiple experimental conditions by measuring the expression of cellular markers within stimulation groups, between stimulation groups and treatment conditions using paired statistical tests. For flow cytometry data, D’Agostino and Pearson’s omnibus normality test was used to check the distribution of the data. If data proved to be normally distributed, the repeated measures one way ANOVA with Sidak’s multiple comparisons test or the paired t test was used to assess if the means of the groups were different, and significance was concluded if p < 0.05. If the data were not normally distributed, the Wilcoxon Signed Rank test or Friedman test was used to test if the means of groups are different, again with p < 0.05 considered significant. For multi-plex assay data, one-way ANOVA with Tukey *post-hoc* correction (Statistica, Quest Software, Elliot Management Group) was used to assess whether the means of groups were different. A p-value less than 0.05 was considered significant.

## Results

### MDSC From TB Patients Suppress T Cell Responses

**Figure 1:**
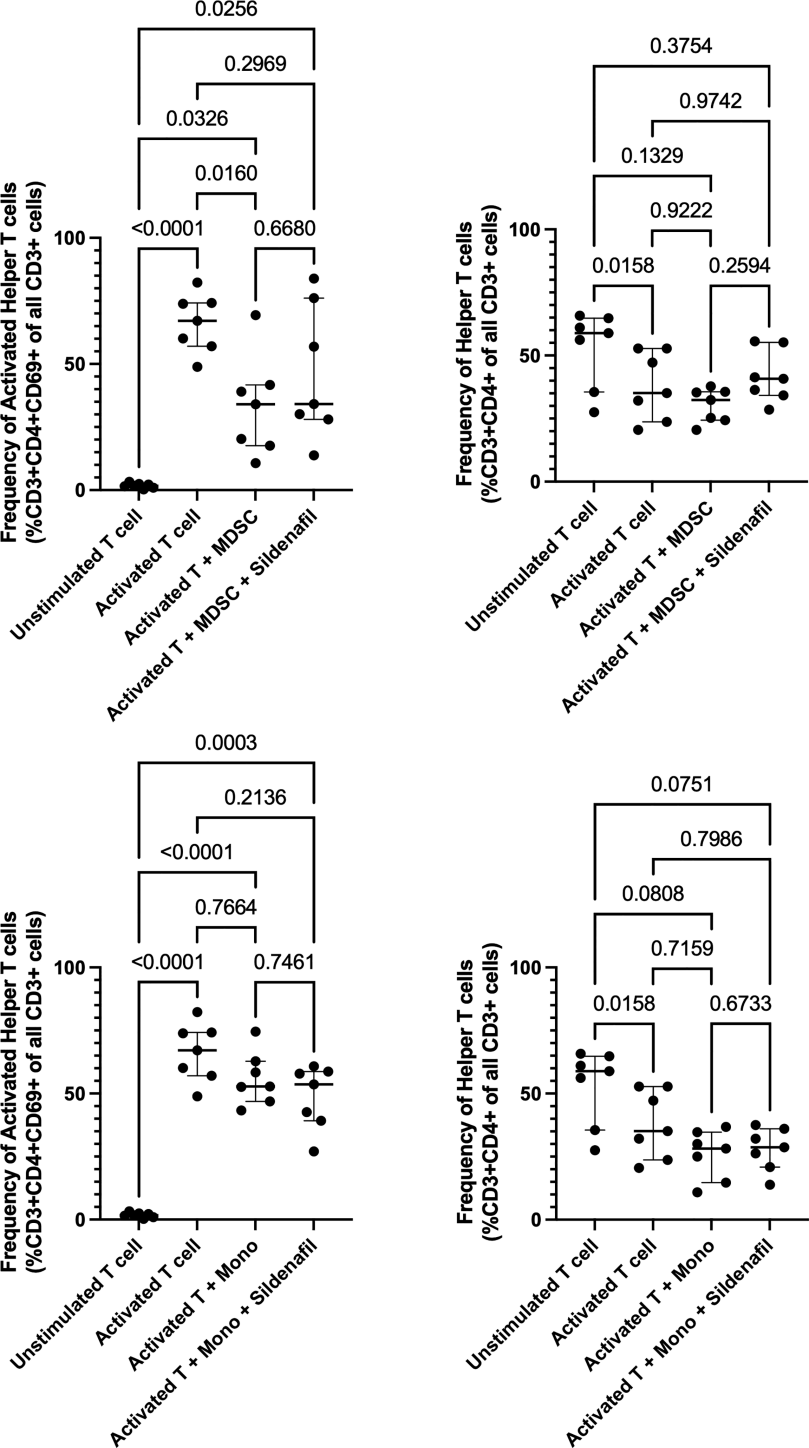
Effect of MDSC on Helper T cell and Helper T cell subset responses. Frequency of Helper T cells (CD3+CD4+) and Helper T cell subsets from in vitro T cell co-culture of autologous T cells (CD3+ cells), MDSC (HLA-CD33+ cells) and control monocytes (HLA+ cells) isolated from PBMC of individuals with active TB disease (n = 7). Plots show individual T cell replicates. Panels **(A-D)** D’Agostino & Pearson test measured normality. Repeated measures one way ANOVA with Sidak’s multiple comparisons test (parametric data) and Friedman test (non-parametric data). P<0.05 was considered significant.

Here, we set out to assess the ability of Sildenafil to counteract MDSC-mediated T cell suppression in freshly isolated PBMCs. Following T cell activation, measured by an increase in CD69 expression on CD3+ T cells, the frequency of activated helper T cells significantly increased (p<0.0001, **Figure 1A**). MDSC suppressed helper T cell activation (p=0.0160, **Figure 1A**). Sildenafil treatment failed to restore MDSC-mediated suppression of helper T cell activation (**Figure 1A**). Coculture of isolated unstimulated T cells in the presence of MDSC did not alter the frequency of helper T cells (**Figure 1B**), but a significant decrease was seen following T cell activation (p=0.0158, **Figure 1B**). We also confirmed that the suppression observed was indeed specific to the MDSC enriched population by testing the ability of the control monocyte population from the same participants to replicate this effect (**Figures 1C, D**). The control monocyte population also did not suppress T-cell activation (**Figure 1C**). Taken together, these data confirm our previous study by showing that MDSC from TB patients supress T-cell responses (11, 16). Sildenafil, however, was unable to counteract MDSC-mediated T cell suppression at the concentration tested.

### Sildenafil Supplementation Does Not Affect MDSC Frequency or Function

**Figure 2:**
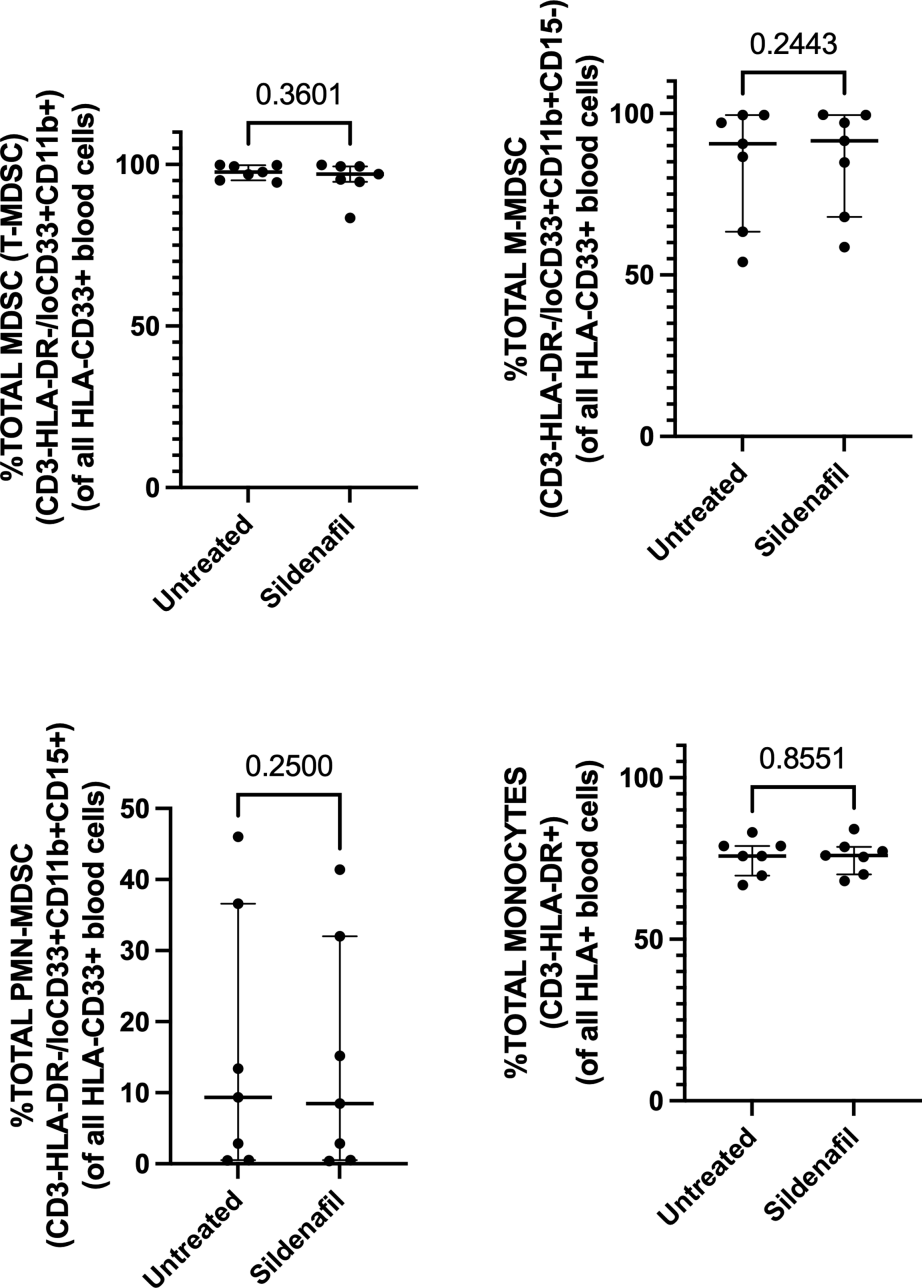
Effect of Sildenafil on the frequency of **(A)** Total MDSC, **(B)** M-MDSC, **(C)** PMN-MDSC, **(D)** Control Monocytes, gated as a frequency of MACS isolated HLA-CD33+ or HLA+ cells, from in vitro cultures of MDSC (HLA-CD33+ cells) and Control Monocytes isolated from PBMC of individuals with active TB disease (n = 7). Plots show individual TB patient data. D’Agostino and Pearson normality Test. Paired t test (parametric data) and Wilcoxon matched pairs signed rank test (non-parametric data). Median with interquartile range indicated on plots. P<0.05 was considered significant.

**Figure 3:**
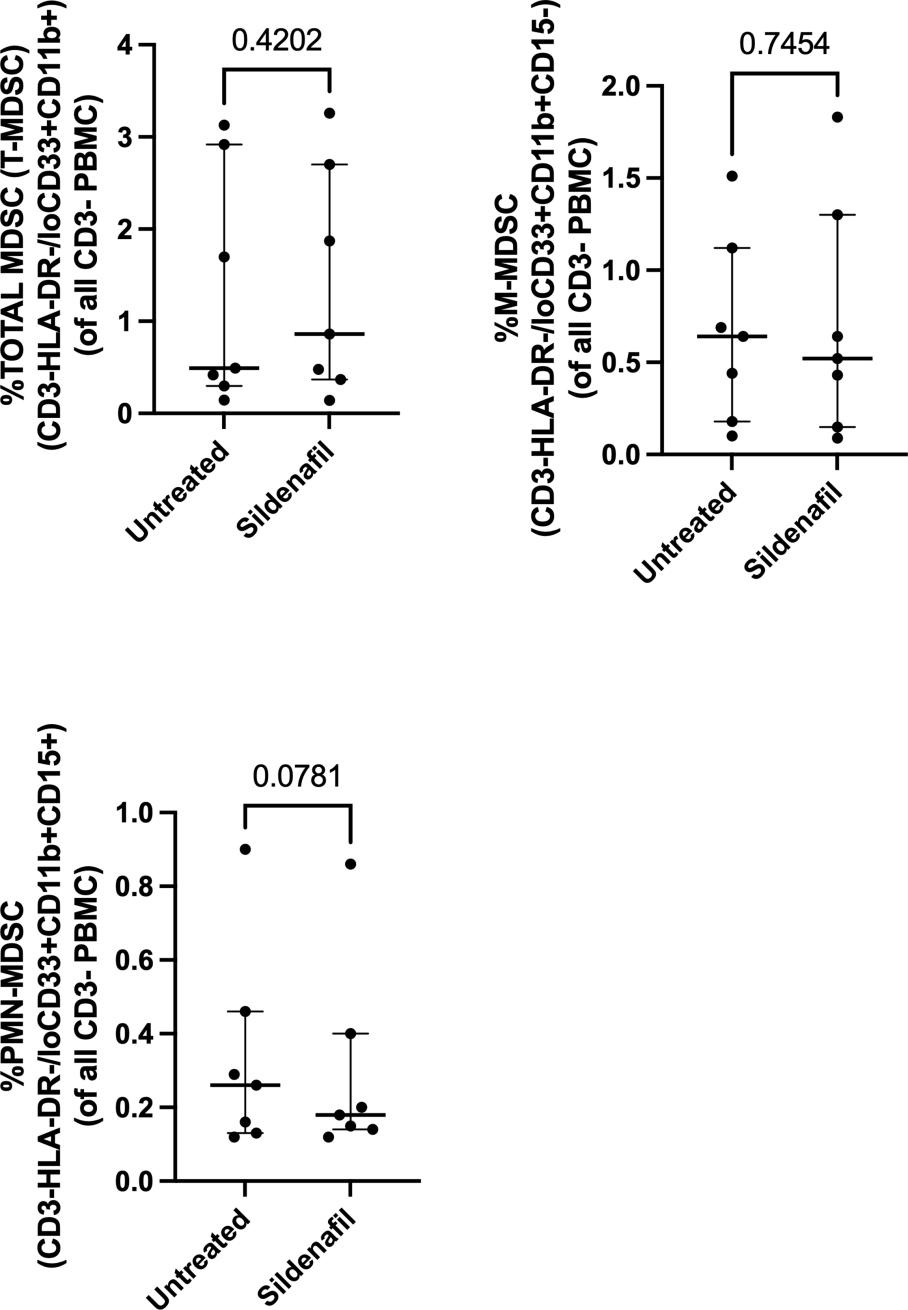
Effect of Sildenafil on the frequency of **(A)** Total MDSC, **(B)** M-MDSC, **(C)** PMN-MDSC, gated as a frequency of PBMC, from in vitro cultures of MDSC (HLA-CD33+ cells) and Control Monocytes isolated from PBMC of individuals with active TB disease (n = 7). Plots show individual TB patient data. D’Agostino and Pearson normality Test. Paired t test (parametric data) and Wilcoxon matched pairs signed rank test (non-parametric data). Median with interquartile range indicated on plots. P<0.05 was considered significant.

Next, we investigated whether *in vitro* treatment of Sildenafil would affect MDSC and control monocyte frequencies. We found >90% enrichment of MDSC (CD3-HLA-DR-/loCD33+CD11b+) (**Figure 2A**) and 75% enrichment of control monocytes (CD3-HLA-DR+) following MACS isolation (**Figure 2D**). Furthermore, we found that M-MDSC comprised 90.6% (Range 54%-99.5%) of the total MDSC population (**Figure 2B**) and 0.64% (Range 0.1%-1.51%) of the PBMC fraction (**Figure 3B**). PMN-MDSC comprised 9.4% (Range 0.49%-46%) of the total population (**Figure 2C**) and 0.26% (Range 0.12%-0.9%) of the PBMC fraction (**Figure 3C**). Following MACS isolation of MDSC and control monocytes from PBMC of TB patients, each cell population was treated with or without 10 uM Sildenafil for 48 hours. We found that Sildenafil treatment had no effect on the frequency of total MDSC, M-MDSC, PMN-MDSC (**Figures 2A-C**) or control monocytes (**Figure 2D**) in the absence of T cells. Taken together Sildenafil had no effect on the frequency of MDSC or control monocytes and M-MDSC dominate the total MDSC population.

### Sildenafil Supplementation Does Not Affect T Cell or MDSC Cytokine Production

**Figure 4:**
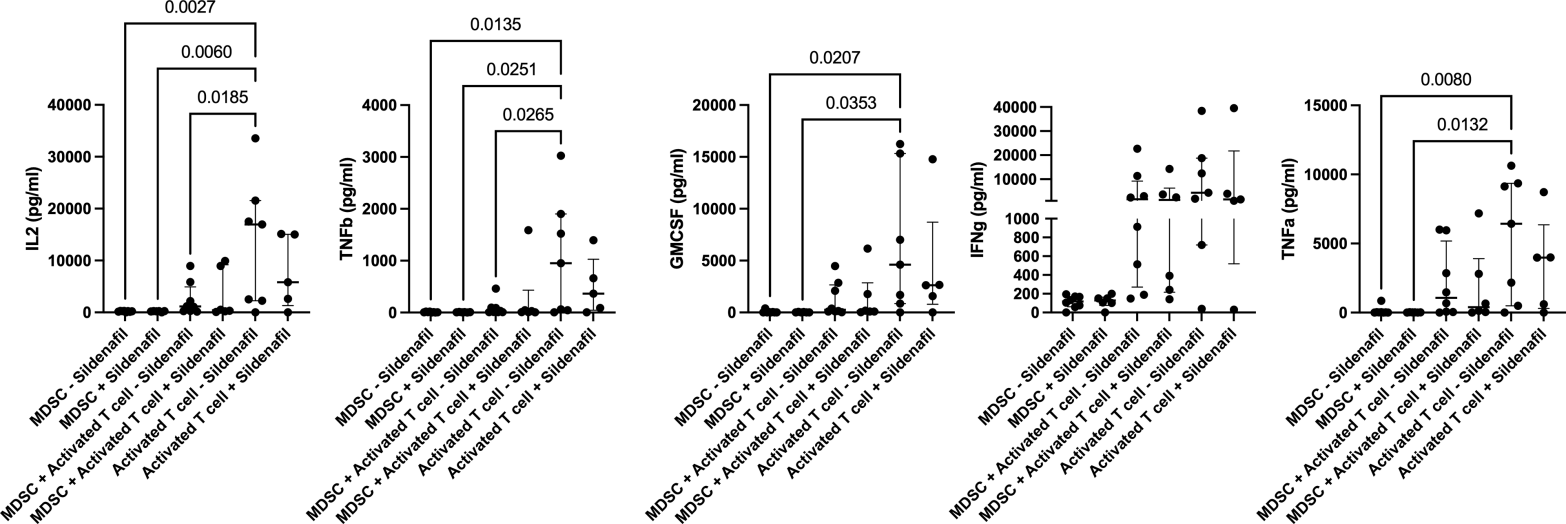
Sildenafil supplementation does not affect T cell or MDSC cytokine production (IL2, TNFb, GMCSF, IFNg, TNFa). Analyte concentration (pg/ml) was measured for various culture combinations (MDSC only, MDSC-T cell coculture, and T cell only), under differing stimulation combinations (Unstimulated and anti-CD3CD28 bead Activation) with or without Sildenafil treatment, by multiplex analysis. Plots show analyte concentration in culture supernatant. One-way ANOVA with Tukey post-hoc correction. Median with interquartile range indicated on plots. P<0.05 was considered significant. Pairwise comparisons which were ns are not shown on plot.

Finally, we investigated whether the MDSC-mediated T-cell suppression shown in our flow cytometry data was also observed in the cellular co-culture supernatant; and whether Sildenafil treatment affected MDSC and T cell cytokine production. MDSCs significantly suppressed T cell production of IL-2 (p=0.0185, **Figure 4A**) and TNFb (p=0.0265, **Figure 4B**) during co-culture in the polyclonally activated condition, without Sildenafil treatment. No MDSC-mediated suppression of T cell cytokine production was observed for GMCSF, IFNg or TNFa (**Figures 4C–E**, respectively). Furthermore, we found that 10uM Sildenafil treatment had no effect on T cell and MDSC cytokine production **Figure 4**; **Supplementary Figures 1**, **2**. Taken together, MDSC suppress T cell cytokine production but Sildenafil supplementation does not restore T cell cytokine production in the presence of MDSC. Sildenafil supplementation also does not affect T cell or MDSC cytokine production.

## Discussion

This study investigated pharmacological modulation of MDSC and the subsequent effect on T-cell immune reactivity, specifically T-cell activation. Strong evidence on the potential of Sildenafil to reverse MDSC immunosuppressive function in cancer and TB mouse models supported Sildenafil treatment as candidate agent for restoring anti-TB host immunity *via* MDSC regulation. We assessed T cell, MDSC and control monocyte phenotype and function following *in vitro* coculture with or without Sildenafil treatment. We confirmed our previous studies (11, 16) by showing that freshly isolated MDSC from TB patients supress autologous non-specifically activated T-cell responses.

Inconsistent with previous reports, we found no restoration of T cell responses when cultured in the presence of MDSC and treated with 10uM Sildenafil. Serafini et al. challenged BALBc mice with a transplantable colon carcinoma, treated with 20mg/kg/24h Sildenafil and observed 50-70% delayed tumour outgrowth. They also isolated tumour derived MDSC, cultured them with T cells and assessed proliferation with or without 50mg/ml Sildenafil treatment and found significant restoration of T cell proliferation (58). The concentration used in the Serafini study was seven thousand four hundred and sixty seven times more concentrated than the concentration used in our cultures (74667uM vs 10uM). The concentration used in our cellular cultures was informed by the peak plasma concentration of Viagra (1uM), when dosed at 50mg. With the intent to see maximum effect, we increased the concentration to 10x the peak plasma concentration following T cell viability testing at various Sildenafil concentrations (**Appendix 1**). 10uM Sildenafil treatment had no negative effect on T cell viability following 48-hour treatment in culture. Future studies should investigate optimised Sildenafil timing and dosage as this could reduce MDSC-mediated T cell suppression, favouring the pro-inflammatory immune response and resulting in increased clearance of *M.tb*. Another important consideration for future research would be the effect of pre-treatment of MDSC with Sildenafil and subsequent removal of Sildenafil from culture, to evaluate the direct effect of Sildenafil-modulated MDSC on T-cell responses. Future investigations should assess the effect of increased Sildenafil concentration, as 10uM Sildenafil did not increase cGMP concentration of MDSC (**Supplementary Figure 3**). This could explain the lack of restoration of T cell responsiveness when treated with Sildenafil in MDSC-T cell cocultures. This is a limitation of the study, and we recommend that future investigations titrate Sildenafil concentrations to achieve the expected increase in cGMP concentrations. Further, the *in vitro* system being used in this investigation may not fully replicate how Sildenafil affects immune function in an *M.tb-*infected host, future investigations should consider this to avoid dealing with a narrow aspect of possible outcomes. Moreover, an increased cohort size should be considered for future investigations. This study was limited by a cohort of 7 treatment-naïve, newly diagnosed, TB participants. The use of non-specific stimulators rather than stimulation with *M.tb* antigens is also limitation of the study as they may mask more subtle differences in modified MDSC function. Future investigations should consider stimulation with *M.tb* purified protein derivatives.

Furthermore, we investigated Sildenafil’s effect on the frequency of MDSC and control monocytes. Sildenafil had no effect on the frequency of total MDSC (**Figures 2A**, **3A**) or the frequency of MDSC subsets, M-MDSC and PMN-MDSC (**Figures 2B**, **3B**), with M-MDSC frequencies contributing to 90.6% of Total MDSC and making up 0.64% of the PBMC fraction. PMN-MDSC contribute 9.4% of the Total MDSC fraction in TB patients and make up 0.26% of the PBMC fraction. Consistent with the MDSC data, Sildenafil had no effect on the frequency of control monocytes (**Figure 2D**). This finding is consistent with our data in **Appendix 1B** and the literature, where Sildenafil had a lack of influence on the cytotoxicity of mononuclear cells (CD8^+^, NKT, and NK cells) (61). Taken together, Sildenafil, at the concentration tested here, had no effect on the frequency of innate immune cells.

In addition to flow cytometric phenotyping, we used multiplex analysis to investigate MDSC and T cell cytokine production within co-culture supernatants. We also investigated whether Sildenafil supplementation influences these cells’ ability to produce cytokines. Our data showed that MDSC suppress T cell production of IL-2 and TNFb, two important pro-inflammatory cytokines with critical roles in the immune response to *M.tb*. This finding is consistent with previous publications within our group (11, 16) and literature (62). However, Sildenafil treatment at 10uM did not restore T cell cytokine responses in the presence of MDSC nor did it influence the cytokine production capacities of MDSC or T cells.

If Sildenafil were to be added as supplementary HDT to the anti-TB treatment, testing of drug–drug interactions between TB medication and Sildenafil would be prudent. Thus far, it has been shown that both Sildenafil and first-line TB drugs (isoniazid, rifampicin, pyrazinamide, ethambutol, and rifabutin) share interactions with cytochrome P450 (Cyp3A) (63, 64). In a study by Maiga *et al.* (2012), they utilized cilostazol and Sildenafil, both FDA approved, in combination with rifampin in their *in vivo* experiments, with the rationale being that the same compounds could be tested in humans. They concluded that cilostazol does not reduce the efficacy of rifampin, but this remains to be tested for Sildenafil (60). Dash *et al.* (2018) used computer modelling studies to examine the docking ability of Sildenafil on *M.tb (* 65). They found that according to the “TB-drugome”, the Rv1555 protein is “druggable” with Sildenafil and has the potential to inhibit the electron transport function during anaerobic respiration, but further validation with *M.tb* strains is required to provide more accurate and reliable proof. Conclusive evidence showing no reduction in the efficacy of TB medication in the presence of Sildenafil is still required and should be investigated. Because coinfection between *M.tb*, HIV and Diabetes mellitus (DM) is a significant problem worldwide, particularly in South Africa, the interactions between Sildenafil, antiretrovirals and diabetes medication should also be considered. Sildenafil has been shown to have no significant change on the effect of ARV levels (saquinavir and ritonavir) (66). Sildenafil has also been shown to be well tolerated in Type II DM patients with poor glycemic control and chronic complications (67). Furthermore, combined treatment of Sildenafil and metformin on diabetic rats improved hyperglycaemia, oxidative stress, and hyperlipidaemia induced by streptozotocin compared to administration of metformin or Sildenafil alone (68). Sildenafil could therefore be considered for patients with TB-HIV-DM coinfection.

Taken together, we have validated previous flow cytometric findings, showing that MDSC induced in the context of TB are immunosuppressive. We showed that Sildenafil treatment does not result in a statistically significant increase in the frequency of activated T cells when in the presence of MDSC. Although higher levels of Sildenafil might yield improved results, the current concentration was selected due to the extensive safety and tolerability data available. Sildenafil inclusion into the anti-TB treatment regimen is questionable, but synergistic effects with other HDTs could be investigated, especially considering the heterogeneous immunosuppressive mechanisms employed by this cell population. Further investigations into repurposed drug compounds targeting MDSC frequency or function are warranted.

## Data Availability Statement

The original contributions presented in the study are included in the article/supplementary material. Further inquiries can be directed to the corresponding author.

## Ethics Statement

The studies involving human participants were reviewed and approved by Stellenbosch University Ethics Review Committee (N16/05/070). The patients/participants provided their written informed consent to participate in this study.

## Author Contributions

NP and GW: conceptualization, supervision, reviewing and editing, and funding acquisition. SM, AH, LK, KR, AK, DS, and AG: methodology, resources, and reviewing and editing. VL: investigation, formal analysis, and writing-original draft. All authors contributed to the article and approved the submitted version.

## Funding

This work was supported by the European & Developing Countries Clinical Trials Partnership (EDCTP; CDF1546), National Institute of Health (NIH) International Collaborations in Infectious Disease Research (ICIDR): Biology and Biosignatures of anti-TB Treatment Response (5U01IA115619/03) and South African National Research Foundation SA Research Chair Initiative (SARCHI; 86535).

## Conflict of Interest

The authors declare that the research was conducted in the absence of any commercial or financial relationships that could be construed as a potential conflict of interest.

## Publisher’s Note

All claims expressed in this article are solely those of the authors and do not necessarily represent those of their affiliated organizations, or those of the publisher, the editors and the reviewers. Any product that may be evaluated in this article, or claim that may be made by its manufacturer, is not guaranteed or endorsed by the publisher.

## Supplementary Material

Supplementary Figure 1 Sildenafil supplementation does not affect T cell or MDSC cytokine production (IL3, IL5, IL10, IL17, IP10). Analyte concentration (pg/ml) was measured for various culture combinations (MDSC only, MDSC-T cell coculture, and T cell only), under differing stimulation combinations (Unstimulated and anti-CD3CD28 bead Activation) with or without Sildenafil treatment, by multiplex analysis. Plots show analyte concentration in culture supernatant. Mixed model analysis, One-way ANOVA with Tukey post-hoc correction. P<0.05 was considered significant.

Supplementary Figure 2 Sildenafil supplementation does not affect T cell or MDSC cytokine production (PDGFAA, MIP1a, MIP1b, CD40L). Analyte concentration (pg/ml) was measured for various culture combinations (MDSC only, MDSC-T cell coculture, and T cell only), under differing stimulation combinations (Unstimulated and anti-CD3CD28 bead Activation) with or without Sildenafil treatment, by multiplex analysis. Plots show analyte concentration in culture supernatant. Mixed model analysis, One-way ANOVA with Tukey post-hoc correction Median with interquartile range indicated on plots. P<0.05 was considered significant.

Supplementary Figure 3 10uM Sildenafil treatment does not increase cGMP concentration within MDSC. cGMP concentration (pmol/ml) was measured in culture supernatant of MDSC treated with 10uM Sildenafil for 48 hours. As positive control, cGMP was measured in culture supernatant of PBMC that were stimulated with 5ug/ml PHA for 12 hours. One-way ANOVA with Tukey post-hoc correction. Median with interquartile range indicated on plots. P<0.05 was considered significant.
