# Supplementary material for: Sildenafil, a Type-5 Phosphodiesterase Inhibitor, Fails to Reverse Myeloid-Derived Suppressor Cell-Mediated T Cell Suppression in Cells Isolated From Tuberculosis Patients
Source: Front Immunol. 2022 Jul 22;13:883886. doi: 10.3389/fimmu.2022.883886 (PMC9353143; doi:10.3389/fimmu.2022.883886)
Supplement: Supplementary Figure 1 — Sildenafil supplementation does not affect T cell or MDSC cytokine production (IL3, IL5, IL10, IL17, IP10). Analyte concentration (pg/ml) was measured for various culture combinations (MDSC only, MDSC-T cell coculture, and T cell only), under differing stimulation combinations (Unstimulated and anti-CD3CD28 bead Activation) with or without Sildenafil treatment, by multiplex analysis. Plots show analyte concentration in culture supernatant. Mixed model analysis, One-way ANOVA with Tukey post-hoc correction. P<0.05 was considered significant. [file DataSheet_1.pdf]

Appendices

Appendix 1. Cell Viability

a

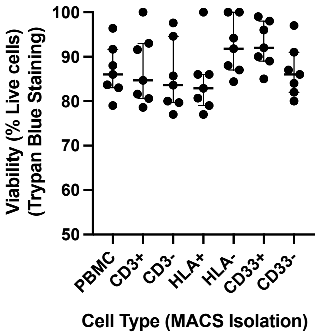

b

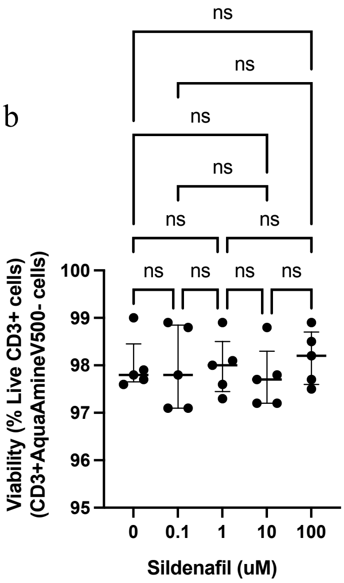

Appendix 2. MACS Isolation Flow Diagram

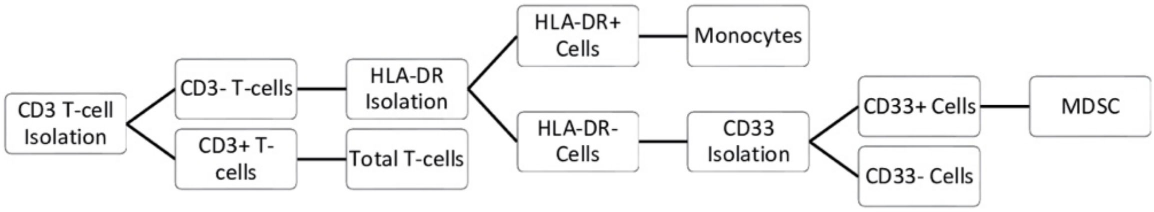

Appendix 3. Co-culture Plate Layout

H20									
Untreated	**M-aT**	**Mo-aT**			**M**	**Mo**	***Sil***		
	**aT**				**T**		***Sil***		
Sildenafil	**M-aT**	**Mo-aT**	***Unstimulated***						
	**aT**		**M**	**Mo**					
			**MT**	**MoT**					
			**T**						
**H20**									

Key:

M: MDSC Mo: Control Monocyte

T: Unstimulated T cell. aT: Stimulated T cell (anti-CD2CD3CD28 beads, Miltenyi T cell Activation Kit)

Appendix 4. Sildenafil dilution

1) Sildenafil (Merck) 5mg/ml Stock.
2) Dilute stock (powder) in 1ml DMSO.
3) Prepared 100 aliquots of 10ul (5mg/ml stock), stored at -20°C
4) Add 90uL RPMI to an aliquot (0.5 mg/mL)
5) [Sildenafil]

 0.5mg/mL = 500 000 ug/L

 500 000 ug/L / 666.7 (Mw) = 749.99uM (concentration in 100 uL)

 X 10 aliquots of Sildenafil = 750uM in 1mL RPMI

 750 uM in 1mL = 100 uM in 7.5 mL

6) Add 6.5 mL RPMI to reach 7.5 mL [100uM Sildenafil]
7) Serial dilution from 100 uM to 0.1 uM

 750 uL from 100uM stock, into 6.75 mL RPMI = 10uM Sildenafil Stock in 7.5 mL

 750 uL from 10uM stock, into 6.75 mL RPMI = 1uM Sildenafil Stock in 7.5 mL

 750 uL from 1uM stock, into 6.75 mL RPMI = 0.1 uM Sildenafil Stock in 7.5 m

Appendix 5a. Gating Strategy (T Cell Panel)

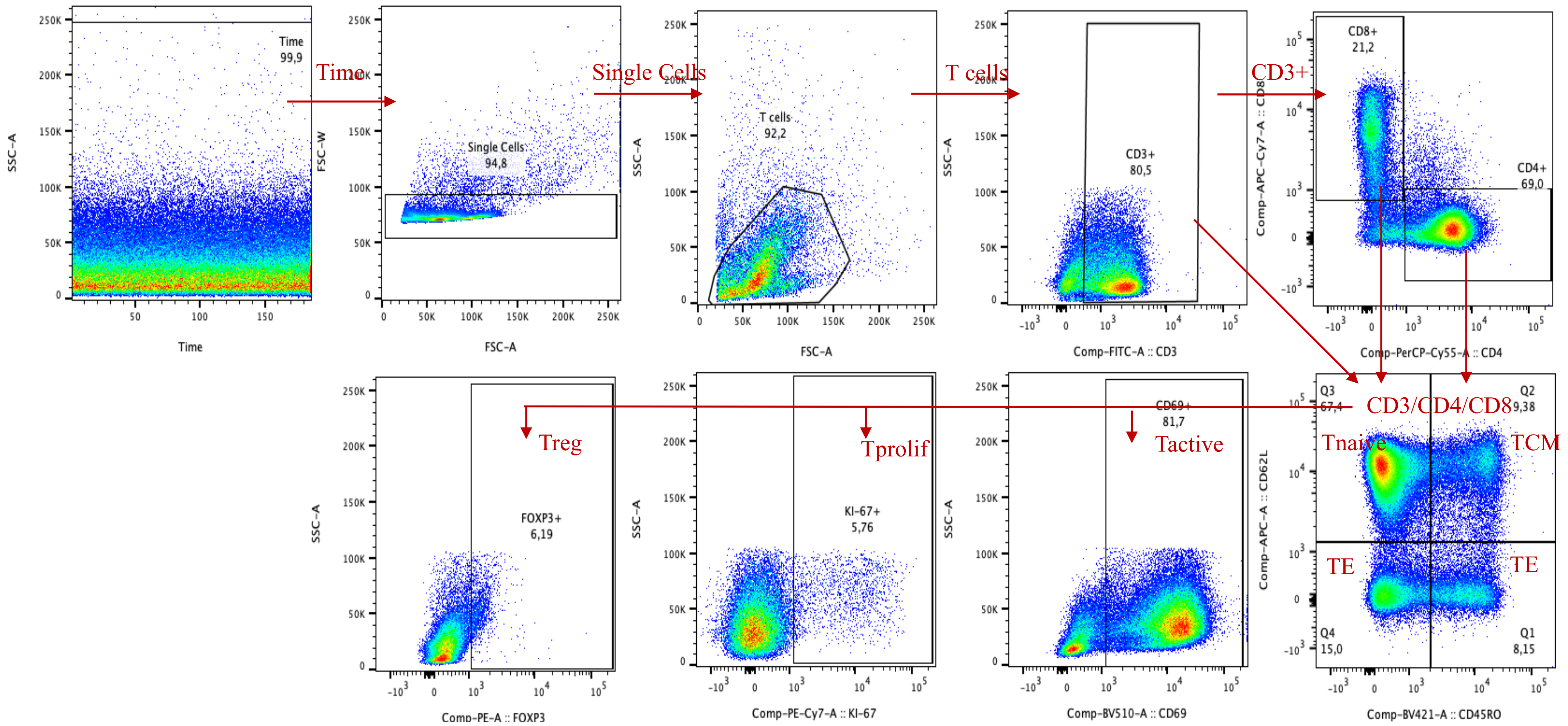

Appendix 5b. Gating Strategy (MDSC Panel)

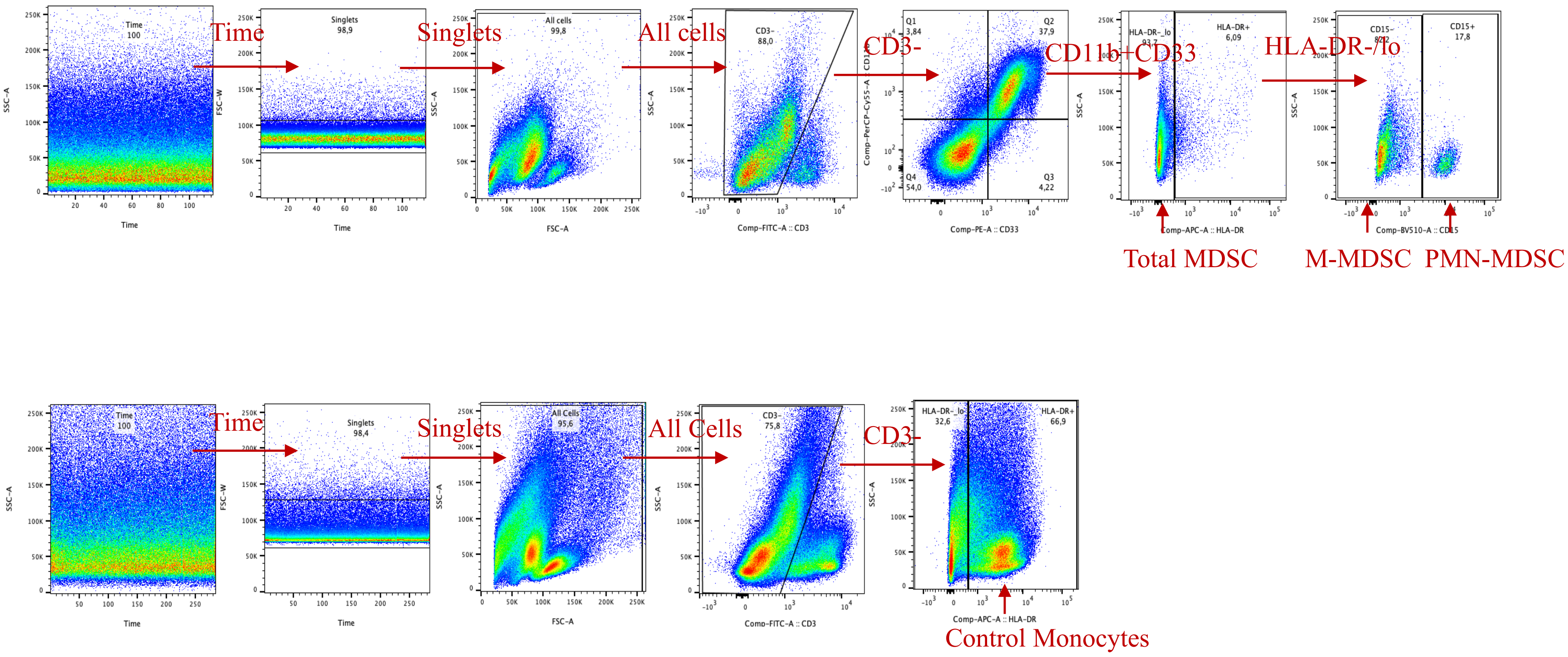

Supplementary Figure 1:

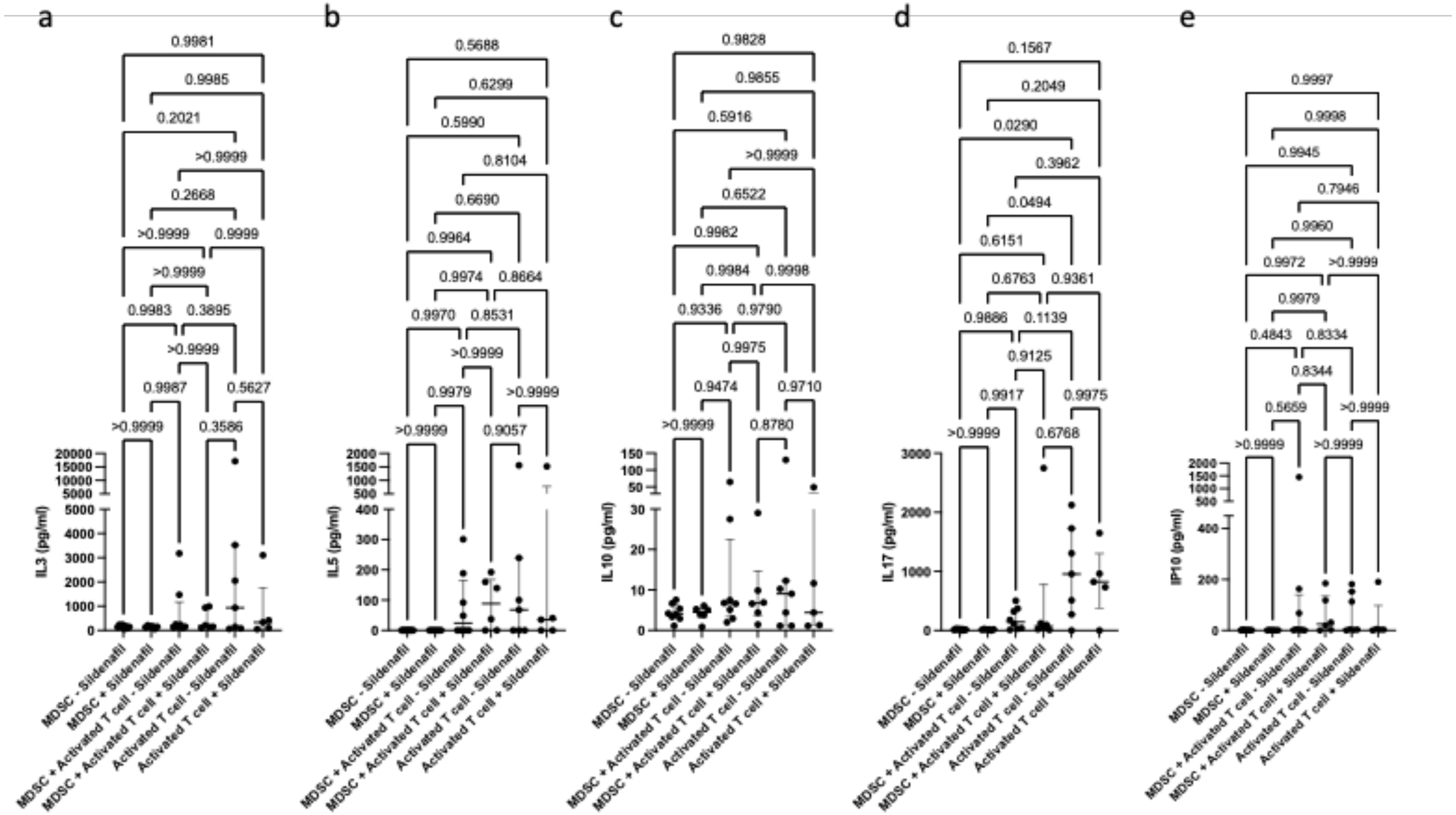

Supplementary Figure 1: Sildenafil supplementation does not affect T cell or MDSC cytokine production (IL3, IL5, IL10, IL17, IP10). Analyte concentration (pg/ml) was measured for various culture combinations (MDSC only, MDSC-T cell coculture, and T cell only), under differing stimulation combinations (Unstimulated and anti-CD3CD28 bead Activation) with or without Sildenafil treatment, by multiplex analysis. Plots show analyte concentration in culture supernatant. Mixed model analysis, One-way ANOVA with Tukey post-hoc correction. P<0.05 was considered significant.

Supplementary Figure 2:

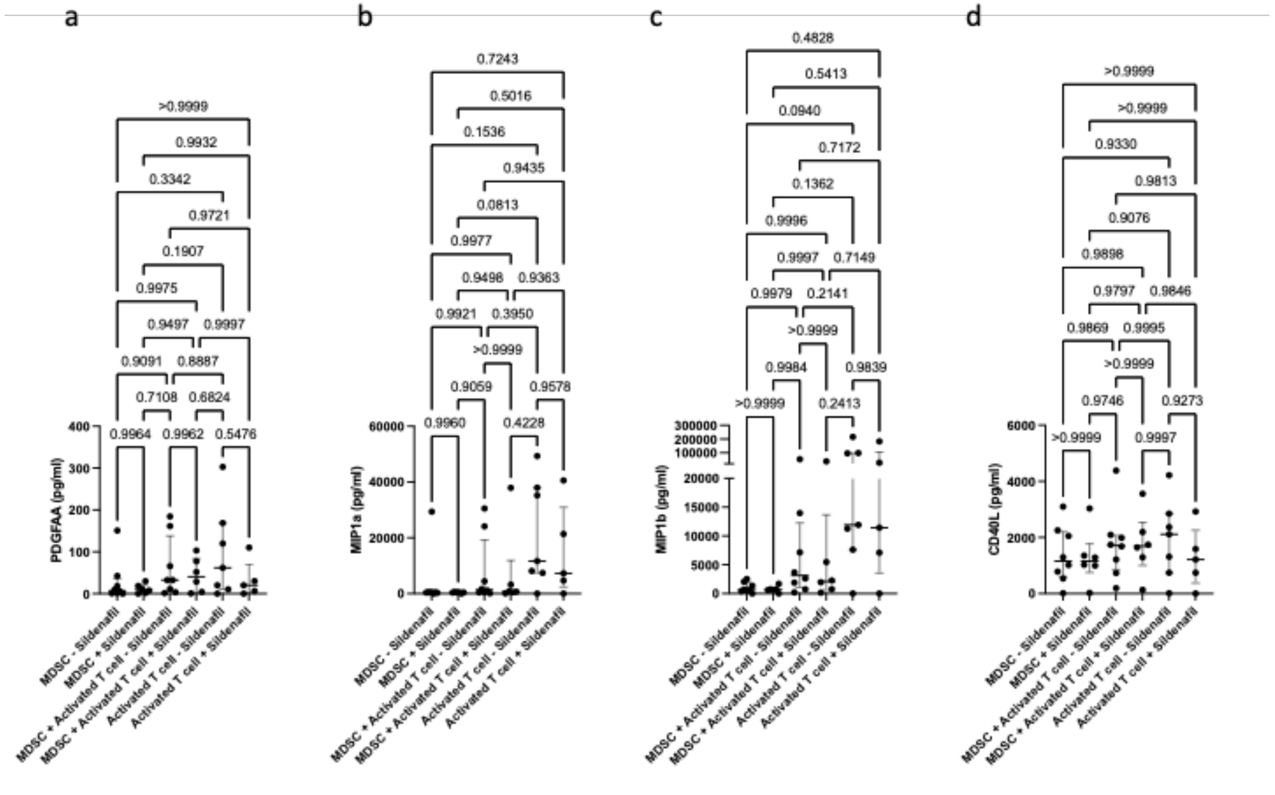

Supplementary Figure 2: Sildenafil supplementation does not affect T cell or MDSC cytokine production (PDGFAA, MIP1a, MIP1b, CD40L). Analyte concentration (pg/ml) was measured for various culture combinations (MDSC only, MDSC-T cell coculture, and T cell only), under differing stimulation combinations (Unstimulated and anti-CD3CD28 bead Activation) with or without Sildenafil treatment, by multiplex analysis. Plots show analyte concentration in culture supernatant. Mixed model analysis, One-way ANOVA with Tukey post-hoc correction Median with interquartile range indicated on plots. P<0.05 was considered significant.

Supplementary Figure 3:

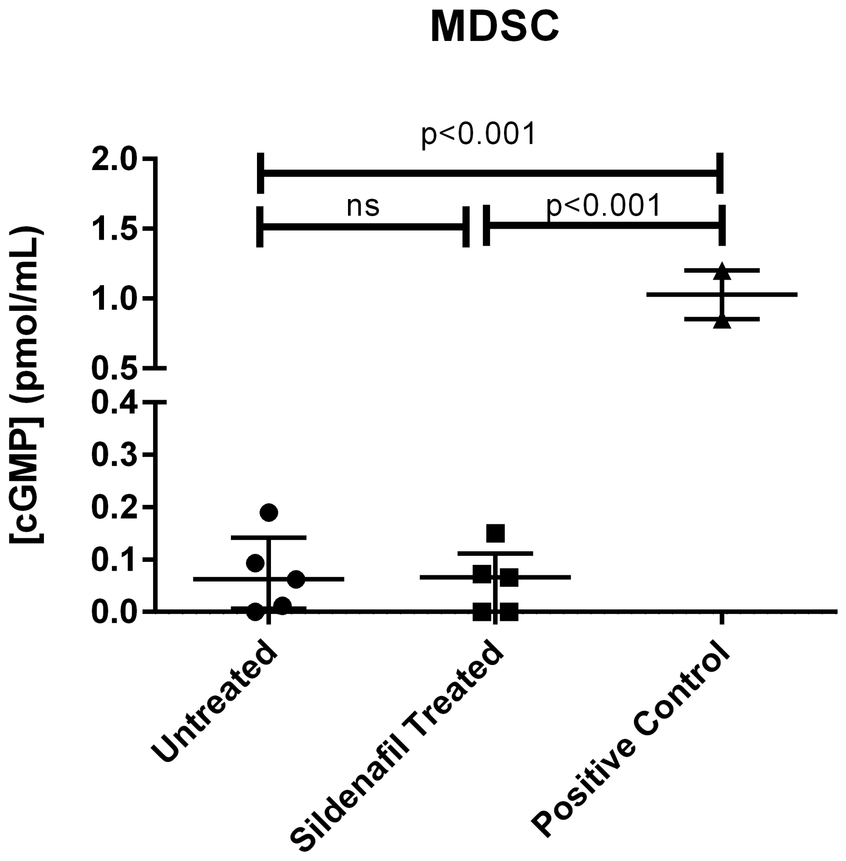

Supplementary Figure 3: 10uM Sildenafil treatment does not increase cGMP concentration within MDSC. cGMP concentration (pmol/ml) was measured in culture supernatant of MDSC treated with 10uM Sildenafil for 48 hours. As positive control, cGMP was measured in culture supernatant of PBMC that were stimulated with 5ug/ml PHA for 12 hours. One-way ANOVA with Tukey post-hoc correction. Median with interquartile range indicated on plots. P<0.05 was considered significant.
